# Cloning and characterization of the *LvCTL* genes encoding C-type lectin from white-leg shrimp ( *Litopenaeus vannamei*)

**DOI:** 10.12688/f1000research.126044.2

**Published:** 2023-09-08

**Authors:** Tran Vinh Phuong, Xuan Huy Nguyen, Nguyen Quang Lich, Quang Tan Hoang, Ngoc Nguyen Tran, Phuoc Ngoc Nguyen, Nguyen Quang Linh

**Affiliations:** 1 Hue University, Hue, 49000, Vietnam; 2 Institute of Biotechnology, Hue University, Hue, 49000, Vietnam; 3 Faculty of Fisheries, Hue University of Agriculture and Forestry, Hue, Thua Thien Hue, 49000, Vietnam; 4 Faculty of Animal Sciences and Veterinary Medicine, Hue University of Agriculture and Forestry, HUE, 49000, Vietnam

**Keywords:** C-type lectin, gene encoding, LvCTL genes, white leg shrimp.

## Abstract

**Background:** Lectins are carbohydrate-binding protein domains. The C-type designates a requirement for calcium for binding. Proteins contain C-type lectin domains that have a diverse range of functions, including cell-cell adhesion, immune response to pathogens, and apoptosis. This study aimed to investigate the characters of LvCTL-encoding genes from white-leg shrimp ( *Litopenaeus vannamei*) in Central Vietnam.

**Methods:** Two PCR products (LvCTL3 and LvCTL4) were cloned and sequenced. The structure and characterization of LvCTL proteins were predicted using bioinformatics tools.

**Results:** The results showed that the *LvCTL3* gene was 444 nucleotides long and 98.87% similar to the published *LvCTL3* gene (accession number: KF156943). The polypeptide sequence had 147 amino acids, which were 97.28% identical to the reference sequence (AGV68681) and the *LvCTL4* gene had a length of 417 nucleotides and homology of 99.52% compared to the published gene (KM387560). The deduced polypeptide sequence had 138 amino acids, and was 100% similar to the reference sequence (AKA64754). The *LvCTL3* had a molecular weight of 16.91 kDa and an isoelectric point (pI) of 4.66, while *LvCTL4* had 15.75 and 4.58 kDa, respectively. The structure prediction results showed that *LvCTL3* and *LvCTL4* had one domain (CTLD), *LvCTL3 *had two α helices and nine β sheets, and *LvCTL4* had two α helices and eight β sheets.

**Conclusions:** Our results provide essential information for the heterologous expression and biosynthesis production of C-type lectins.

## Introduction

C-type lectins (CTLs) are proteins/glycoproteins capable of reversibly binding to carbohydrates by non-covalent bonds without altering their structure ( Zhang *et al.,* 2009). Lectins can bind to cells with glycoproteins or microbial surface glycolipids and are considered pattern recognition receptors (PRR) recognizing and releasing invading microorganisms in the system. The shrimp lectin protein family is very rich and diverse; members of the eight PRR families have been identified in several shrimp species, based on structure and specificity to different sugars of the carbohydrate recognition domain (CRD). These families are lipopolysaccharide and b-1,3-glucan binding proteins (LGBPs), CTLs, galectin, thioester-containing proteins (TEP), fibrinogen-related proteins (FREP), scavenger receptors (SR), down syndrome cell adhesion molecules (DSCAM) and Toll-like receptors (TLR) ( Wang and Wang, 2013). According to form and function, lectins are classified as Calnexin, C-, L-, P-, I-, R-, and S-type lens ( Janeway and Medzhitov, 2002; Zhang *et al.,* 2009). Among the lectins, CTL is the most diverse and well-studied ( Zhang *et al.,* 2009).

CTLs are a group of proteins that play an essential role in many animal biological processes, including cell signaling and pathogen recognition. CTLs are a superfamily of more than 1,000 proteins identified by having one or more well-characterized C-type lectin-like domains (CTLDs). They are divided into 17 subgroups based on their species and domain organization ( Weis *et al.,* 1998; Zelensky and Gready, 2005). CTL also plays an essential role in mammalian innate immunity. The mannose-binding lectin (MBL) is primarily expressed in the liver and released into the plasma, where it recognizes potentially pathogenic bacteria and binds to microbial surfaces. Animals can also activate the complement pathway ( Thiel and Gadjeva, 2009). Similarly, CTLs are also important immune recognition receptors in invertebrates. CTLs in shrimp can currently be divided into three subgroups based on domain composition and organization: those containing only one C-type lectin domain (CTLD), those having two CTLDs, and those containing one CTLD and another part ( Wang and Wang, 2013). Among them, the group containing only one CTLD was more common than the few CTLs in shrimp containing two CTLDs. The presence of two or more binding sites for each lectin molecule allows it to bind to many different cell types. The prevalence of CTLD in invertebrates is much higher than in vertebrates. Typically, CTLD is the seventh most common domain in the *Caenorhabditis elegans* genome, but only the 43 ^rd^ most common domain in the human genome ( Zelensky and Gready, 2005). Shrimp CTLD can bind multiple ligands and constructs inducible expression of CTLs. Recent studies have revealed multiple functions and mechanisms of shrimp CTLs in antimicrobial and antiviral immunity. These protective functions of CTLs in the shrimp species *Fenneropenaeus chinensis* are based on their ability to recognize and bind CTLDs ( Sun *et al.,* 2008). In the framework of this study, we present the results of cloning and characterization of the *LvCTL* genes isolated from white-leg shrimp ( *Litopenaeus vannamei*) in Thua Thien Hue as a prerequisite for the production of recombinant LvCTL proteins for further research.

## Methods

### Ethical approval

Animal use in this study was allowed by Hue University of Animal Ethics Committee with certificate reference number: HUVN0018, April 10 ^th^, 2022. Principal Researcher: Nguyen Quang Linh. All efforts were undertaken to minimize the suffering of animals.

### Animals

White-leg shrimp ( *L. vannamei*) with a weight of ~20 g per head, which were collected from shrimp ponds; the samples were aseptically dissected to collect the hepatopancreas shrimp samples and stored in liquid nitrogen, and used directly for RNA extraction.

### Isolation and cloning of *LvCTL* gene

Total RNA from hepatopancreas samples was extracted using the Gene JET RNA Purification Kit (Thermo Scientific, USA) according to the manufacturer's instructions. Electrophoresis was conducted to check the total RNA obtained on 1% agarose gel with TAE 1X at 70V for 30 min by using Power Pac 3000 (Bio-Rad). The cDNA biosynthesis was performed using Revert Aid First Strand cDNA Synthesis kit (Thermo Fisher Scientific, USA), and the obtained product was used directly for PCR amplification.

The sequences of *LvCTL* encoding genes were obtained from GenBank to design a specific primer ( Table 1). PCR was performed using the following conditions: 95°C for 2 min, and then 40 cycles of 95°C for 1 min, 55°C for 30 s, and 72°C for 1 min, followed by a final extension at 72°C for 7 min. PCR products were confirmed by electrophoresis at 70V for 35 min on 1% agarose gel with TAE 1X stained SafeViewTM Classic (abm, Canada).

**Table 1.** Specific primers for *LvCTL* genes cloning.

Genes	Primers	Sequences (5’-3’)	Product sizes	References
*LvCTLD*	LvCTLD-F/ LvCTLD-R	GACTGCACGGGCGACGAG	888	( Junkunlo *et al.*, 2012)
		TTAGACAGCGGTGACGCAGAG		
*LvCTL3*	LvCTL3-F/ LvCTL3-R	CTCTCCAATCCCATCTCAATC	444	( Li *et al.*, 2014)
		CTATTTCTCACAGATAATG		
*LvCTL4*	LvCTL4-F/ LvCTL4-R	TGTTCTGATGGCTGGCTGG	417	( Li *et al.*, 2015)
		TTAAACCATACAAATGGGATG		
*LvAV*	LvAV-F/ LvAV-R	ACCTTATACGAGAAAAGTG	474	( He *et al.*, 2015)
		TCAAAACTTGTCAGCAGATG		
*LvLdlrCTL*	LvLdlrCTL-F/ LvLdlrCTL-R	GAGTGTACCAACAGGGAC	855	( Liang *et al.*, 2019)
		TCACGCCCTCTCACTGGG		

**Table 2.** Characteristic of the gene encoding LvCTLs.

Characteristics	LvCTL3		LvCTL4	
	Li *et al.* (2014)	This study	Li *et al.* (2015)	This study
Gene full lenghts (bp)	579	-	563	-
Coding segment	492	444	471	417
Polypeptide chain (amino acid)	163	147	156	138
Molecular weight (kDa)	17.9	16.91	-	15.75
Isoelectric point (pI)	4.80	4.66	-	4.58
Domain	1	1	-	1
α helix	-	2	-	2
β strain	-	9	-	8

PCR products were recovered from agarose gels and purified using the GeneJET Gel Extraction kit (Thermo Scientific, USA). Purified PCR products were cloned into pGEM T-easy vector with T4 DNA Ligase for 1 hour at room temperature (Promega, USA), then, the recombinant vectors were transferred into *Escherichia coli* TOP10 bacteria using the heat shock method. The recombinant vectors were selected on LB medium (Luria Bertani Agar, Merck, Germany) supplemented with 50 μg/mL Amp, X-Gal/IPTG. *E. coli* cells carrying the recombinant vector (white colonies) were then inoculated with 5 mL of liquid LB medium supplemented with 50 μg/mL Amp, and DNA plasmids were isolated using the GeneJET Plasmid Miniprep Kit (Thermo Fisher Scientific, USA). The isolated DNA plasmid was examined by 1.0% agarose gel electrophoresis at 70V for 35 min and used for *LvCTL* sequencing ( Ha *et al.,* 2018). The sequence of the *LvCTL* genes was analyzed using the Sanger method (First base Company, Malaysia). The results were checked using BioEdit software and then compared with those published in GenBank using the BLAST tool. A phylogenetic tree of CTL encoding genes was built using MEGA 11 software with the Neighbor-Joining algorithm ( Tamura *et al.,* 2021).

### Characterization and molecular structure of the CTL encoding genes

Characterization of LvCTL proteins was determined using bioinformatics tools, including amino acid sequence translation with Expasy, protein domain using SMART ( Letunic *et al.,* 2021), spatial structure model using Phyre2 ( Kelley *et al.,* 2015), isoelectric point using IPC2 ( Li *et al.,* 2014).

## Results

### Isolating and cloning of the *LvCTL* genes

**Figure 1.**
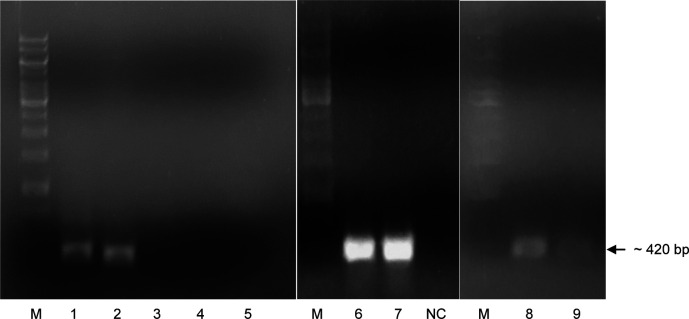
PCR product *LvCTL* genes on agarose gel. M: DNA ladder (GeneRuler ^TM^ 1 kb DNA Ladder, Thermo Scientific, USA). NC: Negative control. 1-5: PCR product with cDNA of *LvCTL3-LvCTL4*- *LvAV-LvCTLD-LvLdlrCTL* genes. 6-7: PCR product of *LvCTL3* gene from 2 different colonies. 8-9: PCR product of *LvCTL4* gene from 2 different colonies.

After PCR with specific primers, the results showed that two DNA fragments were amplified and expressed approximately at 450 and 420 bp in size, respectively. According to the theoretical length of the cDNA fragment of the *LvCTL3* and *LvCTL4* genes. PCR reaction using specific primers with pGEM vector as template was performed, electrophoresis images showed very specific PCR products ( Figure 1), which proved that *LvCTL* genes was successfully attached to pGEM vector T-easy (referred to as the recombinant vector pGEM/LvCTL3 and pGEM/LvCTL4). Therefore, the recombinant vectors were used to analyze the nucleotide sequences. The previous reports indicate several CTLs in some shrimp species, such as: *Litopenaeus vannamei*, *L. schmitti*, *L. setiferus*, *Fenneropenaeus chinensis*, *F. merguiensis*, *Penaeus monodon*, *Marsupenaeus japonicus* and *Macrobrachium rosenbergii* ( Wang and Wang, 2013). We constructed a phylogenetic tree from the deduced LvCTL polypeptide sequences compared with the CTL fragment sequences of other shrimp species. The results show that *LvCTL3* and *LvCTL4* belong to two groups in the dendrogram and our arrangements were similar to the published lines of white-leg shrimp ( *L. vannamei*) but different from other shrimp species ( Figure 6).

### Characteristic determination and molecular structure of the gene encoding CTLs

In our study, the nucleotide sequence of *LvCTL3* PCR product was 444 bp in length, encoding a polypeptide sequence of 147 amino acids. The nucleotide sequence of *LvCTL3* PCR product was 417 bp in length, encoding a polypeptide sequence of 138 amino acids. These were fragment of genes containing the open reading frame (ORF) region cut off the peptide signal, intended to be used to produce recombinant *LvCTL* protein for other studies related to recombinant protein production. For the conserved domain determined using the SMART program, the ORF sequence of *LvCTL3* had one domain (CTLD) from amino acid position 14 to 146. *LvCTL3 *had a molecular weight of 16.91 kDa, and the isoelectric point (pI) was 4.66. The results of spatial structure prediction showed that the *LvCTL3* molecule had two alpha helices (16%) and nine beta strands (28%) ( Figure 7). Meanwhile, the ORF sequence of *LvCTL4* had one domain (amino acid 1 to 137), a molecular weight of 15.75 kDa and a pI is 4.58. LvCTL4 structure had two alpha helices (17%) and eight beta strands (32%) ( Figure 8).

## Discussion

**Figure 2.**
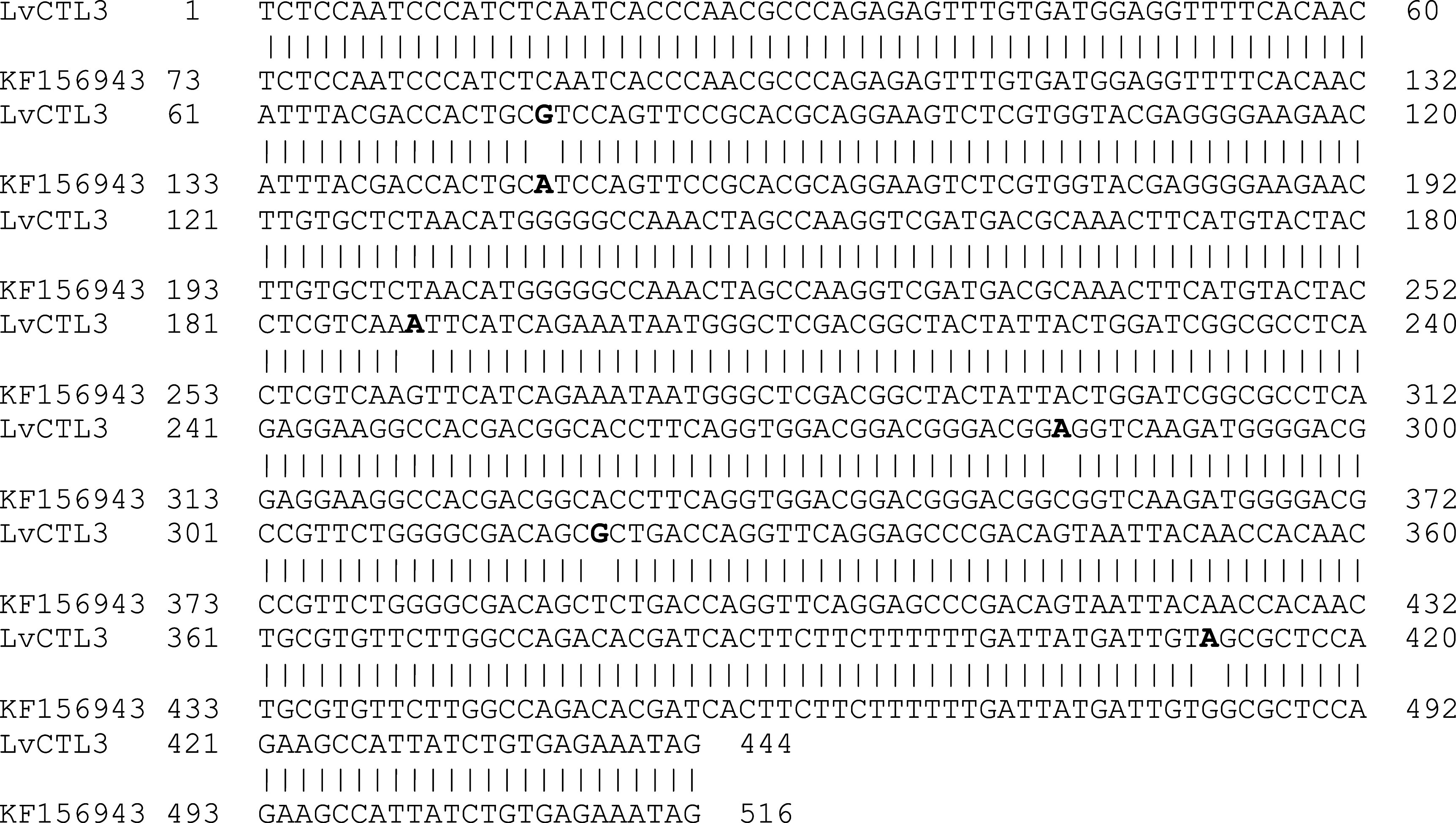
The similarity of the nucleotide sequences of the isolated *LvCTL3* gene and the published *LvCTL3* gene (KF156943).

**Figure 3.**
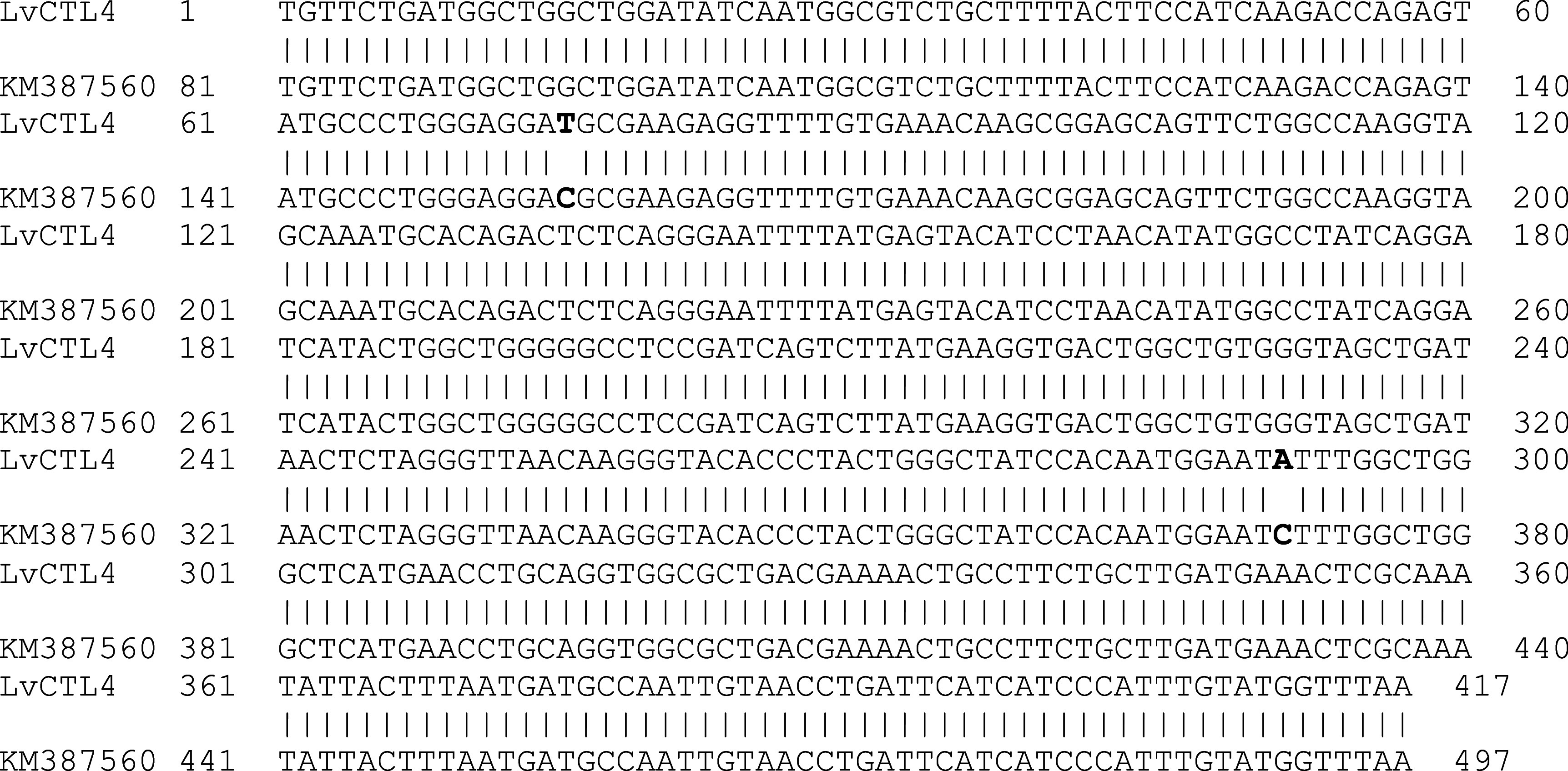
The similarity of the nucleotide sequences of the isolated *LvCTL4* gene and the published *LvCTL4* gene (KM387560).

The results of sequence analysis showed a change in the isolated *LvCTL* genes compared with the published genes. The isolated *LvCTL3* fragment was 444 bp in size (including the stop codon), 98.87% homologous to the published gene (439/444 nucleotides compared with KF156943) ( Figure 2). The change of the *LvCTL3* gene resulted in a difference in the sequence of the encoded polypeptide, the highest similarity obtained was 97.28% (143/147 amino acids) compared with the reference sequence AGV68681. Meanwhile, the *LvCTL4* fragment was 417 bp, 99.52% homologous to the gene of KM387560 (415/417 nucleotides) ( Figure 3). However, the change of the *LvCTL4* gene resulted in no change in the sequence of the encoded polypeptide (100% similarity) compared with the reference sequence (AKA64754).

**Figure 4.**
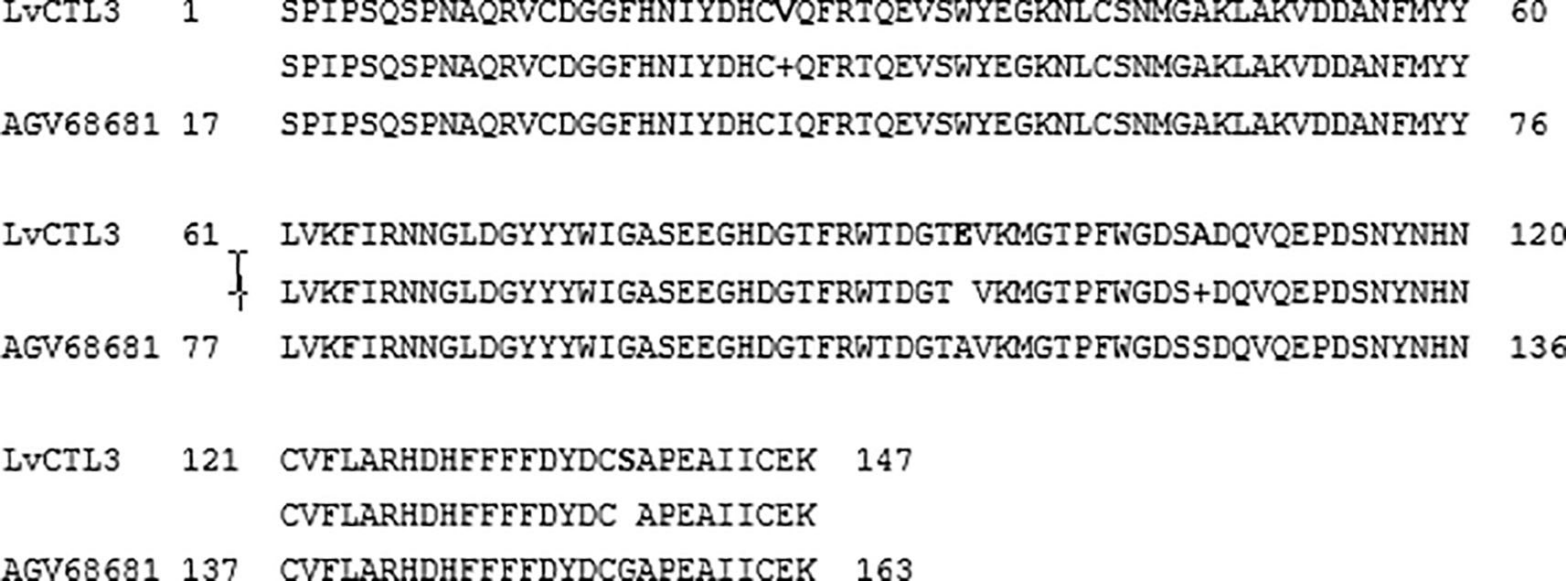
The similarity of the amino acid of the isolated *LvCTL3* gene and the published *LvCTL3* gene (AGV68681).

**Figure 5.**
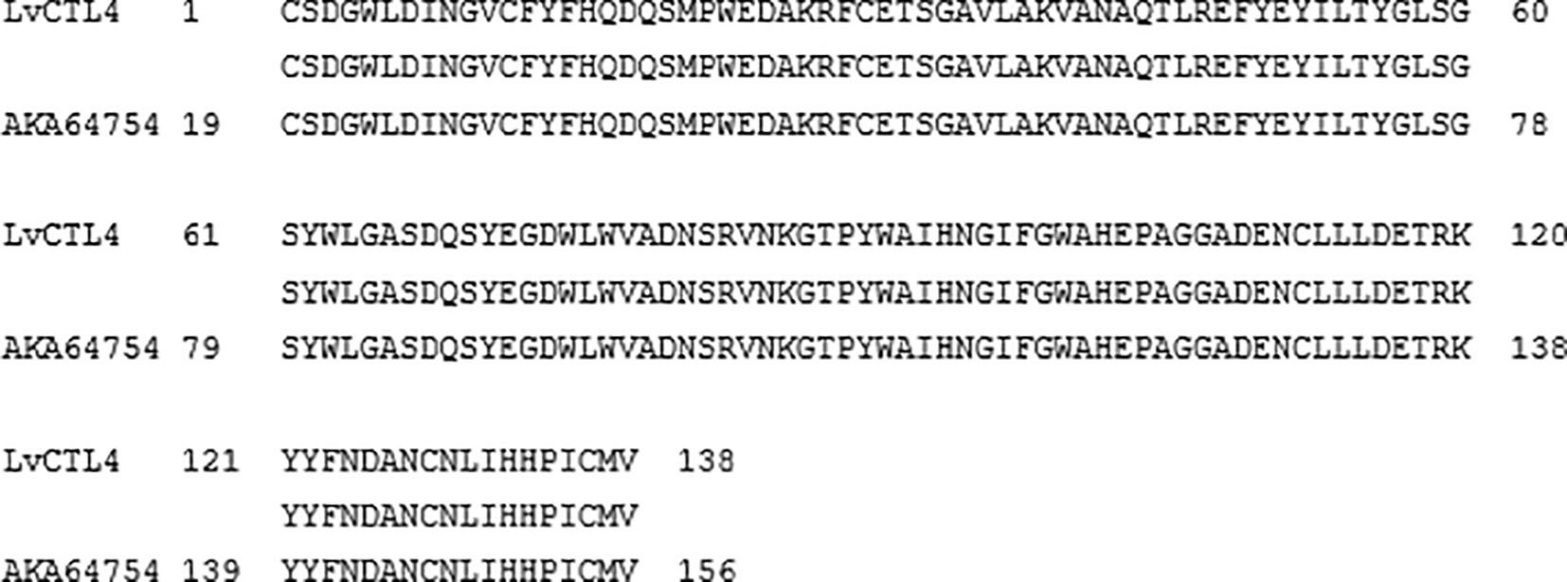
The similarity of the amino acid of the isolated *LvCTL4* gene and the published *LvCTL4* gene (AKA64754).

**Figure 6.**
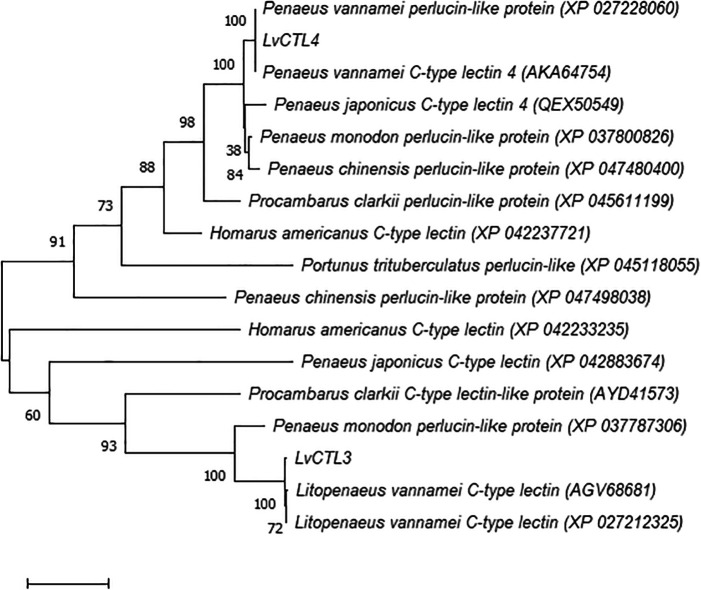
Taxonomy tree LvCTL compared to other published CTLs.

**Figure 7.**
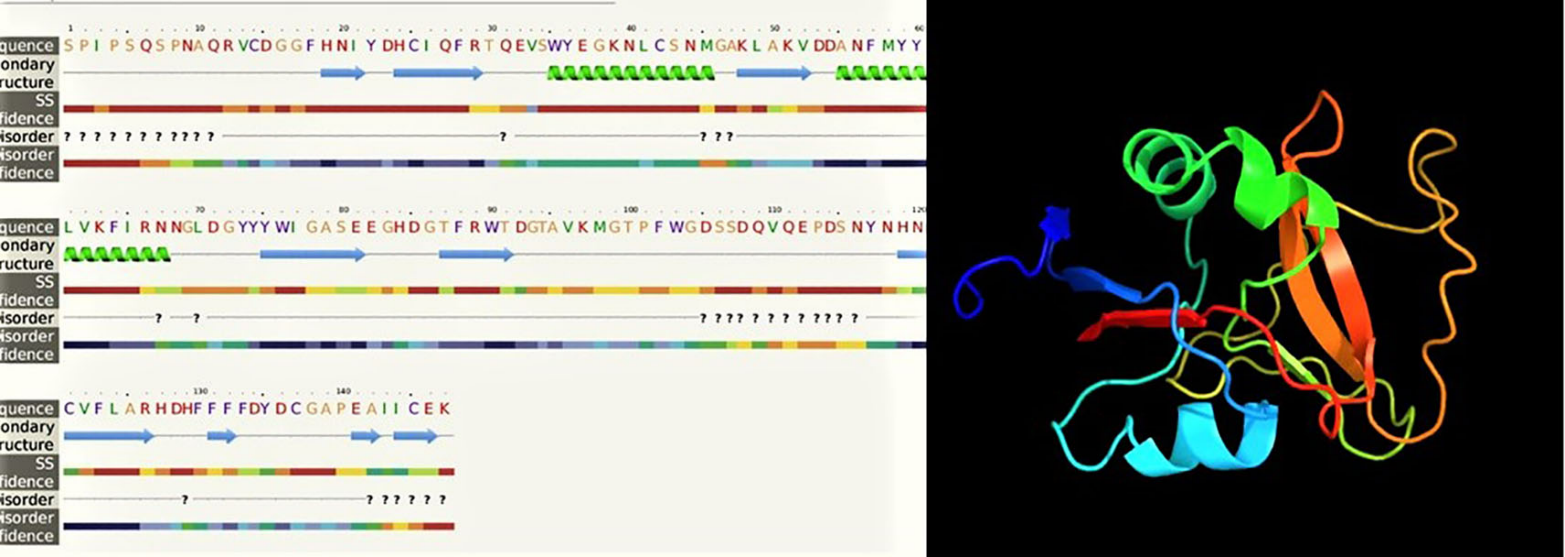
The predicted of LvCTL3 structure.

**Figure 8.**
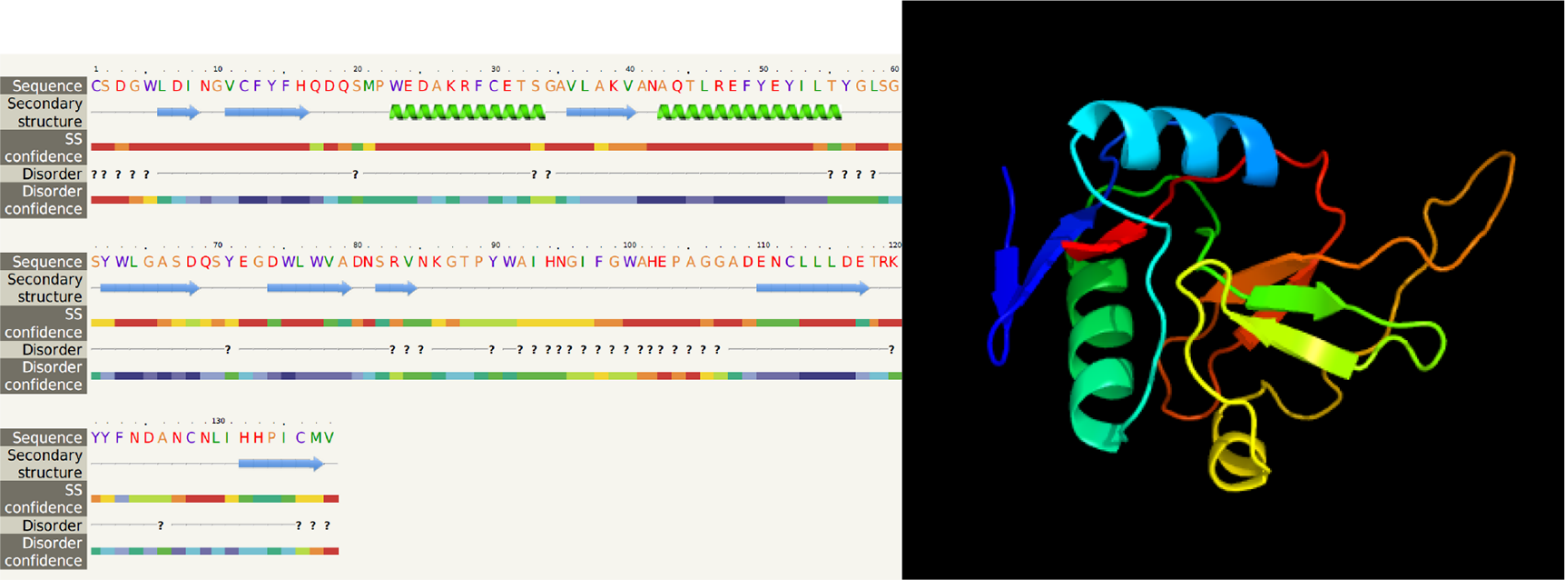
The predicted of LvCTL4 structure.

According to Li *et al.* (2014), the *LvCTL3* gene from shrimp ( *L. vannamei*) has a length of 579 bp, in which the coding segment is 492 bp (from nucleotide positions 25 to 516), encoding a polypeptide chain with a length of 163 amino acids, and has an estimated molecular weight of 17.9 kDa, an estimated pI of 4.80 ( Li *et al.,* 2014). The *LvCTL4* gene, also from *L. vannamei* shrimp, has a length of 563 bp, in which the coding segment is 471 bp (from nucleotide positions 27 to 497). The ORF encoded a protein of 156 amino acids consisting of a single CTLD (residues 19-155) and a putative signal peptide (residues 1-18). Tissue expression analysis showed LvCTL4 was distributed with high levels in the gills, intestine, epithelium and hepatopancreas ( Li *et al.,* 2015). Meanwhile, LvTRAF3 was also cloned and characterized from the shrimp *L.vannamei*; it had a transcript of 3,865 bp, with an ORF of 1,002 bp, and encoded a polypeptide of 333 amino acids, with a calculated molecular weight of 38.6 kDa ( Li *et al.,* 2020).TRAF3 functions as a regulator of innate immune response that involves many cellular processes.

When comparing to the *L. vannamei* genes, the predicted *LvCTL3* sequence resulted in 97.28% similarity to the sequence of AGV68681 or 96.60% to XP_027212325, while *LvCTL4* was 100% similar to the sequence of AKA64754 and XP_027228060. *LvCTL3* and *LvCTL4* were 60.69% (XP_042883674) and 80.43% (QEX50549) homologous to kuruma prawn ( *Penaeus japonicus*), respectively. Compared with black tiger shrimp ( *Penaeus monodon*), *LvCTL3* was 59.18% similar to the protein encoded by XP_037787306 while *LvCTL4* was 88.21% similar to that of XP_037800826. Compared with other crustacean species, the difference in LvCTL sequences was higher. The similarity of the LvCTL3 sequence was only 45.58% identical to the AYD41573 sequence from red swamp crayfish ( *Procambarus clarkii*) or 40.25 % to the XP_042233235 sequence from the American lobster ( *Homarus americanus*). *LvCTL4* was only 59.12% similar to the XP_045611199 sequence from red swamp crayfish, 60.14% to XP_042237721 from the American lobster, or 57.258% to the XP_045118055 sequence from gazami crab ( *Portunus trituberculatus*).

The amino acid sequence alignments of two proteins ( Figure 4 & Figure 5) that may cause protein secondary structure shifts ( Li *et al.*, 2015). The form of a protein is related to its function. Knowledge of protein’s 3D structure is a huge hint for understanding how protein’s work. Although other refinement tools such as homology-modeling tools based on high sequence similiritues provide higher quality structure. However, our 3D structures have been modelled with 99.9% and 99.8% confidence by the single highest scoring template with the 3D structure of LvCTL3 and LvCTL4, respectively. This showed that the function of these proteins is not changed. Protein folding is determined by the physicochemical properties that are encoded in the amino acids. Although most of the predictions were highly accurate, the system that we used is not perfect. Programs such as Alpha Fold will exponentially increase our general understanding of different biological processes. So the 3D structure of these proteins should be further study by another program to provide higher-quality structures. And the simulation of these proteins at pH of shrimp stomach (5.7) should be considered for further experiments.

## Conclusions

In this study, two *LvCTL3* and *LvCTL4* genes from white-leg shrimp were successfully isolated, cloned, and sequenced. The ORF of *LvCTL* sequences had homology levels of 98.87% ( *LvCTL3*, KF156943) and 99.52% ( *LvCTL4*, KM387560 respectively) compared to the published gene. The predicted LvCTL proteins have one conserved domain and are defined as C-type lectins. They can be used for heterologous expression and scale-up production of recombinant C-type lectin in the future to add to the aquatic feed to enhance immunity and prevent disease in shrimp.

## Data Availability

Genbank: C-type lectin [Penaeus vannamei]; Accession number: OP584994, https://www.ncbi.nlm.nih.gov/nuccore/OP584994.1/. Genbank: C-type lectin 4 [Penaeus vannamei]; Accession number: OP584995, https://www.ncbi.nlm.nih.gov/nuccore/OP584995.1/. Figshare: F1000Research Figures, https://doi.org/10.6084/m9.figshare.21428799.v1 ( Linh *et al*., 2022). This project contains the following underlying data:
- 1. *LvCTL3-LvCTL4-LvAV-LvCTLD-LvLdlrCTL *(PCR 5 gene).jpg (Western Blot gel image) - 2. *CTL3*-pcr (Original).jpg (Western Blot gel image) - 3. *CTL4*-pcr (Original).jpg (Western Blot gel image) - 4. bw *LvCTL3-LvCTL4*- *LvAV-LvCTLD-LvLdlrCTL* (Edited).jpg (Western Blot gel image) - 5. *LvCTL3* PCR colony (Edited).jpg (Western Blot gel image) - 6. CTL4 bw - colony (Editted).jpg (Western Blot gel image) - 7. The predicted of LvCTL3 structure.png (Predicted 3D structure of LvCTL3) - 8. The predicted of LvCTL4 structure.png (Predicted 3D structure of LvCTL4) **Protocols.io:** Lectin C gene analysis, https://dx.doi.org/10.17504/protocols.io.x54v9dyk1g3e/v1 ( Phuong *et al.*, 2022). Data are available under the terms of the Creative Commons Attribution 4.0 International license (CC-BY 4.0).
