# Supplementary material for: Cloning and characterization of the LvCTL genes encoding C-type lectin from white-leg shrimp ( Litopenaeus vannamei)
Source: F1000Res. 2024 Jun 17;12:260. Originally published 2023 Mar 10. [Version 3] doi: 10.12688/f1000research.126044.3 (PMC11240081; doi:10.12688/f1000research.126044.3)
Supplement: Peer review history

# Reviewer report: Reviewer response for version 3

**DOI:** 10.5256/f1000research.156535.r295554

**Published:** 2024-08-24

**Authors:** Parin Chaivisuthangkura

**Affiliations:** 1 Srinakharinwirot University, Bangkok, Bangkok, Thailand

This manuscript described the isolation of CTL genes from L. vannamei with the intention to express the recombinant protein in the future. The isolated PCR products, LvCTL3 and LvCTL4 were sequenced, analyzed and the structure was predicted.

1. The designed primers must indicate the nucleotide positions of PCR products to provide the intention of amplification. In Table 1, there were no explanation for each primer pair. Why did you amplify LvCTLD, LvAV,LvdlrCTL and what were those regions? In addition, the successful of PCR amplifications was only occurs with CTL3 and CTL4. What about the other 3 products in Figure 1? The discussion must be added.
2. How many clones of recombinant plasmids were sequenced since there were some discrepancies of nucleotide sequence compared with the GenBank reports? Only one clone is not enough to obtain the correct sequence.
3. Did you use the proofreading enzyme to do the PCR amplification?
4. The first paragraph of results must be rewritten since there were plenty of errors that cause confusion to the audience.
5. The gel picture of Figure 1 must be redone. The marker was not clear and the size of PCR products did not seem to be different. How many percent gel did you use?
6. The first paragraph of discussion must be moved to result section. The explanation of Figure 4 and 5 must be added in the results.
7. Why did you discuss about LvTRAF3? This should be deleted.
8.  The authors should consider adding a Table for the percent similarity of amino acid sequences among CTLs from various shrimp species.

Minor points

 Introduction, L6: use “beta instead of b”

                         L9: Did you mean lectins instead of lens

 Methods

 Animals: Did you mean 20 g per shrimp?

                 Rewrite the whole section since there are plenty of grammatical errors.

 Figure 6:

 Please choose between Litopenaeus vannamei and Penaeus vannamei. Only one term must be used.

 The legend should be “The phylogenetic tree of LvCTL3……..amino acid sequences. 

 Figure 7  legend should be “The predicted LvCTL3 structure”

 Figure 8  legend should be “The predicted LvCTL4 structure”

 Discussion: What did you mean by “the system that we used is not perfect”?

 The underlying data: Do you have any Western blot image in this project? Did you mean DNA gel pictures?

Is the work clearly and accurately presented and does it cite the current literature?

Partly

If applicable, is the statistical analysis and its interpretation appropriate?

Not applicable

Are all the source data underlying the results available to ensure full reproducibility?

Partly

Is the study design appropriate and is the work technically sound?

Partly

Are the conclusions drawn adequately supported by the results?

Partly

Are sufficient details of methods and analysis provided to allow replication by others?

Partly

Reviewer Expertise:

innate immunity in shrimp.

I confirm that I have read this submission and believe that I have an appropriate level of expertise to confirm that it is of an acceptable scientific standard, however I have significant reservations, as outlined above.

# Reviewer report: Reviewer response for version 3

**DOI:** 10.5256/f1000research.156535.r295548

**Published:** 2024-07-15

**Authors:** Ta-Hui Lin

**Affiliations:** 1 Department of Basic Medical Science, Xiamen Medical College, Xiamen, Fujian, China

I confirm that I have read this submitted material and believe that I have the appropriate professional knowledge to verify that it meets acceptable scientific standards. But as mentioned above, I have significant reservations. Some vocabulary and grammar errors in the article need to be corrected.

 The description of the results in the abstract should be written in this way to be correct. "The molecular weight of LvCTL3 is 16.91 kDa and its isoelectric point (pI) is 4.66, while the molecular weight and pI of LvCTL4 are 15.75 kDa and 4.58, respectively."

 At the same time, new articles can be used as a reference for structural and functional analysis.

Is the work clearly and accurately presented and does it cite the current literature?

Yes

If applicable, is the statistical analysis and its interpretation appropriate?

Not applicable

Are all the source data underlying the results available to ensure full reproducibility?

Yes

Is the study design appropriate and is the work technically sound?

Yes

Are the conclusions drawn adequately supported by the results?

Yes

Are sufficient details of methods and analysis provided to allow replication by others?

Yes

Reviewer Expertise:

Biochemistry, Molecular Biology

I confirm that I have read this submission and believe that I have an appropriate level of expertise to confirm that it is of an acceptable scientific standard, however I have significant reservations, as outlined above.

## References

[1] : Molecular Characterization and Expression Analysis of the C-Type Lectin Domain Family 4 Member F in Litopenaeus vannamei against White Spot Syndrome Virus. *Animals (Basel)* .2024;14(8) : 10.3390/ani14081137 10.3390/ani14081137 38672285 PMC11047491

# Reviewer report: Reviewer response for version 3

**DOI:** 10.5256/f1000research.156535.r295546

**Published:** 2024-07-11

**Authors:** Mengqiang Wang

**Affiliations:** 1 Ocean University of China, Qingdao, China

F1KR00CDE F1R-VER156535-R

 C-type lectins are glycan-binding pattern recognition receptors (PRRs) that can bind to carbohydrates on pathogen surfaces, triggering immune responses in shrimp innate immunity. The manuscript, Cloning and characterization of the LvCTL genes encoding C-type lectin from white-leg shrimp ( *Litopenaeus vannamei*), cloned and characterized two C-type lectin genes (LvCTL3 and LvCTL4) from white-leg shrimp, which provided essential information for the heterologous expression and biosynthesis production of C-type lectins. The authors provided detailed procedure of their experiments in Methods section, the laboratory experiments were well designed and executed. The overall experimental design is sound and solid, the results are convincing in general. The current study provides heterologous expression and scale-up production of recombinant C-type lectin in the future to add to the aquatic feed to enhance immunity and prevent disease in shrimp. The manuscript qualifies for indexing.

Is the work clearly and accurately presented and does it cite the current literature?

Yes

If applicable, is the statistical analysis and its interpretation appropriate?

Yes

Are all the source data underlying the results available to ensure full reproducibility?

Yes

Is the study design appropriate and is the work technically sound?

Yes

Are the conclusions drawn adequately supported by the results?

Yes

Are sufficient details of methods and analysis provided to allow replication by others?

Yes

Reviewer Expertise:

Innate immunity in aquaculture.

I confirm that I have read this submission and believe that I have an appropriate level of expertise to confirm that it is of an acceptable scientific standard.

# Response

**Published:** 2024-07-14

**Authors:** Linh Q. NGUYEN

**Affiliations:** Nutritional Disease and System, Hue University, HUE, Thua Thien Hue, Vietnam

Dear Editor and Reviewer 1, 2, 

 Thank you for your approval of our article. We agreed with all your comments and assistance, so version 3 would be nice for publication in the last version.

 Thank you so much; see you soon in the next manuscript.

 Nguyen Quang Linh

# Reviewer report: Reviewer response for version 2

**DOI:** 10.5256/f1000research.147015.r205043

**Published:** 2023-09-29

**Authors:** Hieu Tran-Van

**Affiliations:** 1 Laboratory of Biosensors, Faculty of Biology and Biotechnology, University of Science, Ho Chi Minh, Vietnam

The authors have satisfactorily responded to all my questions and made the necessary changes to the manuscript. Therefore, the manuscript can be accepted for indexing in its current form.

Is the work clearly and accurately presented and does it cite the current literature?

Yes

If applicable, is the statistical analysis and its interpretation appropriate?

Not applicable

Are all the source data underlying the results available to ensure full reproducibility?

Yes

Is the study design appropriate and is the work technically sound?

Yes

Are the conclusions drawn adequately supported by the results?

Yes

Are sufficient details of methods and analysis provided to allow replication by others?

Yes

Reviewer Expertise:

Biotechnology, molecular modelling, immunology

I confirm that I have read this submission and believe that I have an appropriate level of expertise to confirm that it is of an acceptable scientific standard.

# Reviewer report: Reviewer response for version 1

**DOI:** 10.5256/f1000research.138419.r166106

**Published:** 2023-03-28

**Authors:** Hieu Tran-Van

**Affiliations:** 1 Laboratory of Biosensors, Faculty of Biology and Biotechnology, University of Science, Ho Chi Minh, Vietnam

The authors cloned and characterized two *LvCTL* genes encoding C-type lectin from white-leg shrimp ( *Litopenaeus vannamei*). 

 There are points authors should improve before consideration for indexing.

1. Authors should consider using recent protein structure prediction tools such as AlphaFold or homology-modeling tools based on high sequence similarities such as SWISS-MODEL or MODELLER. Other refinement tools should be applied for higher-quality structures.
2. Should model reference proteins of Li et al. publication to give readers a better visualization of amino acid differences that may cause protein secondary structure shifts.
3. The authors didn’t discuss any information about protein structural characteristics. According to a previous study, shrimp stomach pH is approximately 5.7, so maybe authors should consider simulation the protein at that pH for further experiments.
4. Does the deletion in the DNA sequence of LvCTL3 and LvCTL4 in your study might affect its functions?
5. According to your discussion, what is the purpose of building a 3D model of these two proteins?
6. Authors should include amino acid sequence alignments of two proteins to highlight the differences.

Is the work clearly and accurately presented and does it cite the current literature?

Yes

If applicable, is the statistical analysis and its interpretation appropriate?

Not applicable

Are all the source data underlying the results available to ensure full reproducibility?

Yes

Is the study design appropriate and is the work technically sound?

Yes

Are the conclusions drawn adequately supported by the results?

Yes

Are sufficient details of methods and analysis provided to allow replication by others?

Yes

Reviewer Expertise:

Biotechnology, molecular modelling, immunology

I confirm that I have read this submission and believe that I have an appropriate level of expertise to confirm that it is of an acceptable scientific standard, however I have significant reservations, as outlined above.

# Response

**Published:** 2023-04-10

**Authors:** Linh Q. NGUYEN

**Affiliations:** Nutritional Disease and System, Hue University, HUE, Thua Thien Hue, Vietnam

1

 Authors should consider using recent protein structure prediction tools such as AlphaFold or homology-modelling tools based on high sequence similarities such as SWISS-MODEL or MODELLER. Other refinement tools should be applied for higher-quality structures.

 The amino acid sequence alignments of two proteins (Figure 4 & Figure 5) may cause protein secondary structure shifts (Li *et al.*, 2015). The form of a protein is related to its function. Knowledge of a protein’s 3D structure is a huge hint for understanding how proteins work. Although other refinement tools, such as homology-modelling tools based on high sequence similarities, provide higher-quality structure. However, our 3D structures have been modelled with 99.9% and 99.8% confidence by the single highest-scoring template with the 3D structure of LvCTL3 and LvCTL4, respectively. This showed that the function of these proteins is not changed. Protein folding is determined by the physicochemical properties that are encoded in the amino acids. Although most of the predictions were highly accurate, our system is imperfect. Programs such as Alpha Fold will exponentially increase our general understanding of different biological processes. So the 3D structure of these proteins should be further studied by another program to provide higher-quality structures. And the simulation of these proteins at the pH of the shrimp stomach (5.7) should be considered for further experiments.

 We added **Fig. 4**. The amino acid similarity of the isolated *LvCTL3 *gene and the published *LvCTL3* gene (AGV68681) and **Fig. 5**. The similarity of the amino acid of the isolated *LvCTL4* gene and the published *LvCTL4* gene (AKA64754) and discussed in the text (purple colour)

 2

 Should model reference proteins of Li et al. publication to give readers a better visualization of amino acid differences that may cause protein secondary structure shifts.

 3

 The authors didn’t discuss any information about protein structural characteristics. According to a previous study, shrimp stomach pH is approximately 5.7, so maybe authors should consider simulation the protein at that pH for further experiments

 4

 Does the deletion in the DNA sequence of LvCTL3 and LvCTL4 in your study might affect its functions?

 5

 According to your discussion, what is the purpose of building a 3D model of these two proteins?

 6

 Authors should include amino acid sequence alignments of two proteins to highlight the differences.

 7

**Another revision**

1. Added 3rd affiliation of the first author: Tran Vinh Phuong ^1^^, ^^3^
2. Updated another affiliation (Ex: Faculty of Fisheries, University of Agriculture and Forestry, Hue University, Thua Thien Hue, 49000, Vietnam)
3. Underlying data

Updated 2 new links, Genbank, to replace old ones.

https://www.ncbi.nlm.nih.gov/nuccore/OP584994.1/ and

https://www.ncbi.nlm.nih.gov/nuccore/OP584995.1/

1. Writing mistake:

This project contains the following underlying data:

**-** 2. *CTL3*-pcr (Original).jpg (Western Blot gel image)

**-** 3. *CTL4*-pcr (Original).jpg (Western Blot gel image)

1. Grant Information

[Tran Vinh Phuong] was funded by the Master, PhD Scholarship Programme of Vingroup Innovation Foundation (VINIF), [VINIF.2022.TS097]. This study was partly supported by the Hue University Strong Research Group (NCM.DHH.2022.05) and by the Vietnam Ministry of Science and Technology (project code: DTDLCN.56/22)

 Red colour

# Response

**Published:** 2023-09-13

**Authors:** Vinh Phuong Tran

Dear Reviewer; 

 We would like to thank the reviewer for your comments on our manuscript. We have carefully considered your suggestions in revising our manuscript, and we believe that the revised manuscript is improved and suitable for publication. Detailed responses to the comments are presented in this document

 1. Authors should consider using recent protein structure prediction tools such as Alpha Fold or homology-modeling tools based on high sequence similarities such as SWISS-MODEL or MODELLER. Other refinement tools should be applied for higher-quality structures

**Responses**: 

 Although other refinement tools such as homology-modeling tools based on high sequence similarities provide higher quality structure. However, our 3D structures have been modelled with 99.9% and 99.8% confidence by the single highest scoring template with the 3D structure of LvCTL3 and LvCTL4, respectively. Programs such as Alpha Fold will exponentially increase our general understanding of different biological processes. So the 3D structure of these proteins should be further study by another program to provide higher-quality structures

 2. Should model reference proteins of Li et al. publication to give readers a better visualization of amino acid differences that may cause protein secondary structure shifts.

**Responses**: 

 Thanks for your value comment. We used the model reference proteins of Li et al. 2015 to give readers a better visualization of amino acid differences that may cause protein secondary structure shifts in the revised version

 3. The authors didn’t discuss any information about protein structural characteristics. According to a previous study, shrimp stomach pH is approximately 5.7, so maybe authors should consider simulation the protein at that pH for further experiments

**Reponses**

 We agreed and added this sentence "the simulation of these proteins at pH of shrimp stomach (5.7) should be considered for further experiments" in the discussion of the new version.

 4. Does the deletion in the DNA sequence of LvCTL3 and LvCTL4 in your study might affect its functions?

**Responses**

 The form of a protein is related to its function. Knowledge of protein’s 3D structure is a huge hint for understanding how protein’s work. Although other refinement tools such as homology-modeling tools based on high sequence similarities provide higher quality structure. However, our 3D structures have been modelled with 99.9% and 99.8% confidence by the single highest scoring template with the 3D structure of LvCTL3 and LvCTL4, respectively. This showed that the function of these proteins is not changed. Protein folding is determined by the physicochemical properties that are encoded in the amino acids

 5. According to your discussion, what is the purpose of building a 3D model of these two proteins?

**Responses**

 The purpose of building a 3D model of these two protein was explained in the discussion of the new version as read bellowed:

 The amino acid sequence alignments of two proteins (Figure 4 & Figure 5) that may cause protein secondary structure shifts ( Li *et al.*, 2015). The form of a protein is related to its function. Knowledge of protein’s 3D structure is a huge hint for understanding how protein’s work.

 6. Authors should include its amino acid sequence alignment of two proteins to highlight the differences.

**Responses**

 We added **Figure 4**. The similarity of the amino acid of the isolated *LvCTL3 *gene and the published *LvCTL3* gene (AGV68681) and **Figure 5**. The similarity of the amino acid of the isolated *LvCTL4* gene and the published *LvCTL4* gene (AKA64754) and discussed in the text.

 Thank you very much for your consideration of this manuscript.

 Yours sincerely,
